# Moving from legality to reality: how medical abortion methods were introduced with implementation science in Zambia

**DOI:** 10.1186/s12978-017-0289-2

**Published:** 2017-02-16

**Authors:** Tamara Fetters, Ghazaleh Samandari, Patrick Djemo, Bellington Vwallika, Stephen Mupeta

**Affiliations:** 1 Ipas, 300 Market St., Suite 200, Chapel Hill, NC USA; 2 Gillings School of Public Health, University of North Carolina at Chapel Hill, Chapel Hill, NC USA; 3 Regional program Manager, Francophone Africa, Lusaka, Zambia; 4 Department of Obstetrics and Gynecology, Consultant Obstetrician and Gynecologist University of Zambia School of Medicine, Lusaka, Zambia; 5 National Reproductive Health Specialist, UNFPA, Lusaka, Zambia

**Keywords:** Abortion, Zambia, Medical abortion, Implementation science, Scale up, Postabortion care, Reproductive health

## Abstract

**Background:**

Although abortion is technically legal in Zambia, the reality is far more complicated. This study describes the process and results of galvanizing access to medical abortion where abortion has been legal for many years, but provision severely limited. It highlights the challenges and successes of scaling up abortion care using implementation science to document 2 years of implementation.

**Methods:**

An intervention between the Ministry of Health, University Teaching Hospital and the international organization Ipas, was established to introduce medical abortion and to address the lack of understanding and implementation of the country’s abortion law. An implementation science model was used to evaluate effectiveness and glean lessons for other countries about bringing safe and legal abortion services to scale. The intervention involved the provision of Comprehensive Abortion Care services in 28 public health facilities in Zambia for a 2 year period, August 2009 to September 2011. The study focused on three main areas: building health worker capacity in public facilities and introducing medical abortion, working with pharmacists to provide improved information on medical abortion, and community engagement and mobilization to increase knowledge of abortion services and rights through stronger health system and community partnerships.

**Results:**

After 2 years, 25 of 28 sites provided abortion services, caring for more than 13,000 women during the intervention. For the first time, abortion was decentralized, 19% of all abortion care was performed in health centers. At the end of the intervention, all providing facilities had managers supportive of continuing legal abortion services. When asked about the impact of medical abortion provision, a number of providers reported that medical abortion improved their ability to provide affordable safe abortion. In neighboring pharmacies only 19% of mystery clients visiting them were offered misoprostol for purchase at baseline, this increased to 47% after the intervention. Despite progress in attitudes towards abortion clients, such as empathy, and improved community engagement, the evaluation revealed continuing stigma on both provider and client sides.

**Conclusions:**

These findings provide a case study of the medical abortion introduction in Zambia and offer important lessons for expanding safe and legal abortion access in similar settings across Africa.

## Plain english summary

Although abortion is legal in Zambia, very few women know they have the right to have an abortion or know where to seek this care. Unsafe abortions have caused unnecessary deaths in Zambia even among women who have the right to a safe and legal abortion. In this national study we used the opportunity to introduce abortion with medication and improve understanding of the law and abortion care in and around 28 health facilities in the country.

In this study routine statistics from more than 13,000 women were collected from health facilities and combined with survey data from women seeking abortions, trained health care workers and community members to illustrate the results of the national efforts to introduce and scale up abortion services.

This study is an example of tackling a difficult issue, involving a wide range of different groups and partners and proposing a national model. This study also provides an example of work and lessons for other countries around Africa.

## Background

Although abortion is technically legal in Zambia, the reality is far more complicated. Zambia has what has been called a “paper law” with numerous barriers to care at both the policy and implementation levels [1]. Unsafe abortion remains a very real problem - causing death and disability across the country. Although evidence on the incidence and consequences of unsafe abortion in recent years is scarce, numerous studies from the late 1990s identify the methods used across the country, such as ingesting toxins like detergent and inserting cassava sticks in the cervix [2–4]. While information and utilization of legal abortions is becoming more common, most experts feel that unsafe abortion is persisting [5–7]. Between 2003 and 2008 in Zambia’s major hospitals, almost one-third of all gynecologic admissions were due to complications of unsafe abortion, researchers estimated that 6 in every 1,000 of these women died as a result of their complications [8].

The impact of recent maternal health interventions on the incidence of unintended pregnancy and unsafe abortion remains unclear. Early childbearing is still very common, almost 60% of Zambian women have borne a child by age 19 [9]. Rural young women in poverty bear the brunt of morbidity from early childbearing, facing twice the risk of complications during pregnancy as older women and severely limited opportunities for continued education that help lift women and their families from poverty [9]. Regardless of education, socioeconomic status or place of residence, most Zambian women have more children than they originally wanted [9].

Until recently, legal abortion services have not been widely available in health centers or hospitals, compelling women to continue to turn to illegal providers and unsafe methods and confirming that just the existence of a law, with insufficient political will or guidance for implementation may not be effective in improving maternal health [6, 10–12]. Unsafe abortion – the termination of an unintended pregnancy either by persons lacking the necessary skills or in an environment lacking the minimal medical standards, or both – contributes to 8-13% of global maternal mortality [13, 14]. Although Zambia’s Maternal Mortality Ratio (MMR) declined in the preceding 7 years from 591 to 398 per 100,000 births in the 2014 Demographic and Health Survey, it remains high for the African continent; as many as 30% of these deaths could be the result of unsafe abortion according to an audit of maternal deaths at the University Teaching Hospital (UTH) in Lusaka [5].

Medical abortion (MA) using the World Health Organization’s recommended mifepristone and misoprostol combined regimen, is a highly effective method of pregnancy termination, with low complication rates up to and even after 9 weeks of gestation [15–17]. The use of medications offers women a non-surgical method of induced abortion, and has been proven to be highly acceptable to both clients and providers [18, 19]. Particularly in low-resource settings, with limited health care access and few trained surgical abortion providers, MA can increase safe abortion access, and decrease maternal morbidity and mortality associated with unsafe abortion by providing a safe and effective non-surgical option. However, at the time of this study, mifepristone was registered in only two African countries - Tunisia and South Africa; since 2009 only five additional African countries have registered mifepristone for importation - Zambia, Ghana, Mozambique, Ethiopia and Kenya [20]. Restrictive abortion laws, the expense of the medication, and reluctance by policymakers to shepherd mifepristone to broader markets and tackle policies necessary for wider distribution have contributed to few developing countries making this drug available.

In 2008, concerned with alarming maternal death rates and faced with daunting Millennium Development Goals (MDGs), the Ministry of Health (MOH) supported a national strategic assessment of unsafe abortion in the country. Findings revealed high demand for safe abortion in the country, the existing use of a wide variety of unsafe and traditional abortifacients, persistent high levels of provider stigma, logistical barriers to accessing safe abortion for women across the country and lack of specific provisions for youth [21]. On the heels of this assessment, Ipas, an international non-governmental organization (NGO) working in the field of reproductive health and rights, in collaboration with the Ministry of Health (MOH) and the University Teaching Hospital, conducted a comprehensive pilot project to introduce medical abortion (MA) with mifepristone and misoprostol in Zambia and to demonstrate a model for strengthening and scaling up safe abortion services to the extent allowed by the law. The project evaluation was designed using an implementation science model to document the factors and components that could be used to move a national comprehensive abortion care (CAC) program from limited to extensive service provision for replication in other parts of the continent.

This paper first describes the process and results of galvanizing access to medical abortion in an environment where abortion has been legal for many years, but provision of safe services has been severely limited. Secondly, results are presented to highlight the successes and the challenges of bringing medical abortion to an operationally complex context, a health system being rebuilt after enormous investment in HIV prevention and treatment, using implementation science to document the results of 2 years of implementation. Finally, these findings not only provide a case study of medical abortion in Zambia, but also offer important lessons and recommendations for expanding access to safe abortion in similar settings across Africa.

### Establishment of legal abortion in Zambia

Although weak in implementation, Zambia has among the most liberal abortion policies of any Sub-Saharan African country. The Termination of Pregnancy (TOP) Act of 1972 permits abortion in Zambia under the following circumstances: the pregnancy causes risk to the life of the pregnant woman; risk of injury to the physical or mental health of the pregnant woman; risk of injury to the physical or mental health of any existing children of the woman, greater than if the pregnancy were terminated; or if there is substantial risk of fetal malformation. Moreover, the law states that if the continuance of a pregnancy would involve great risk, account may be taken of the pregnant woman's environment or of her age [22]. Further amendments to the Penal Code have allowed for abortion in cases of rape and incest.

The 1972 TOP Act of Zambia contained cumbersome requirements that had to be instituted before a termination could be performed [1]. The TOP Act did not reference gestational limits for an abortion but the MOH later issued regulations for provider authorization through viability (28 weeks of gestation) [23]. The onerous regulations were established under the guise of safeguarding against potential abuses of the law. To this end the law stated that a doctor’s decision to perform an abortion had to be co-signed by two other physicians, one of whom had to be a specialist, before the procedure could be initiated. The law also stipulated that only licensed physicians could perform the procedure and only in facilities designated by government as might be considered appropriate in the United Kingdom but were unrealistic in Zambia where health infrastructure, training and physicians were in short supply. Unfortunately, the regulations now codified in the law, essentially created an impossible standard for implementation [1]. With fewer than 2 physicians for every 10,000 people in the country, even when women have a willing and skilled provider, many women, especially in rural areas, are forced to seek unsafe abortions because they can’t navigate the health system and get the permissions required to have a legal abortion in time to terminate their pregnancies [8, 24]. The Zambian law eliminated any role for midlevel providers - health care workers such as nurses, midwives and medical officers, who have a more restricted scope of practice than physicians - in abortion care without a new law or ministerial guidance overriding it. African midlevel health workers currently provide most of the induced or postabortion care in Africa [25–28].

### Abortion access in Zambia, 1970s-2000s

Although Zambia’s TOP Act was ratified in 1972, very little was done in the post-legalization era to inform the public or improve access to services. The law itself was passed quietly, and was not followed by technical guidance to facilitate implementation; even today, most legislators and judges are unclear about the nuances of the TOP Act [11]. Furthermore, dilation and curettage remained the outdated standard of abortion care for much of the three decades following the passage of the Act, well after the World Health Organization (WHO) technical guidance on abortion technologies recommended manual vacuum aspiration (MVA) and medication for induced abortion. In the early 1990s limited training for legal terminations was introduced in the country with the material support of NGOs and with USAID support. [5, 29–31] However, due to lack of funding and sustainable support from the government, exhaustive resource and infrastructure needs and a loss of health workers during the early years of the AIDS crisis, access to legal abortions never devolved past the University Teaching Hospital (UTH), by the mid 1990’s the UTH was the only facility providing induced abortion services in the country [29, 31, 32].

In the 1990s, due to persistent lack of access to contraceptive methods, and virtually no health system support for abortion provision, women continued to suffer the consequences of unsafe abortions. Alarmingly, many of these procedures were unlawfully performed by untrained providers or traditional practitioners, who benefited from women’s lack of knowledge regarding legal abortion and their inability to access licensed care [33].

In this milieu, some efforts were made to improve access to PAC services. In 1992, the Zambian government restructured the health care system to be more decentralized, moving human and material resources to the purview of the districts. At the same time, the reforms introduced an “essential package” of health care services aimed at addressing the most pressing needs of Zambians. Reproductive health was highlighted under the new reforms, and USAID and Zambian government partners assessed the situation for improving post-abortion care in the country [31]. The introduction and decentralization of safe abortion and PAC services was suggested using manual vacuum aspiration, a technology and service which requires the same skill set as the provision of induced abortion, to eliminate the outdated use of dilatation and curettage in the country. The assessment team did not make strong recommendations about the TOP Act that has hindered access to TOP in rural areas where most care is provided my midwives and nurses and physicians are uncommon [31].

Unfortunately, the timing of PAC reforms coincided with the most devastating period of the HIV epidemic in Zambia. During the 90s the epidemic in Zambia peaked at 16.5% HIV prevalence in the general population, which significantly impacted the population in all conceivable ways. The spread of the disease devastated the health care work force, as doctors, nurses and even policy makers across the country succumbed to their illnesses [34]. The epidemic itself - and the resultant thinning of human resources for health – as well as the restructuring and resources required to rapidly scale up voluntary counselling and testing for HIV, prevention of mother-to-child transmission efforts and antiretroviral treatment throughout the country, shifted the focus of health care towards basic efforts to sustain the population, leaving little for other initiatives.

### 2008 strategic assessment of unsafe abortion

In the new millennium, attention in Zambia was refocused on improving maternal health outcomes, largely due to the Millennium Development Goals. Zambia was tasked with reducing deaths from 591 to 162 per 100,000 live births by 2015 [35]. Although that goal was ultimately proven to be beyond reach, the increased attention to maternal health spurred a renewed examination of unsafe abortion issues.

In response to this problem, in 2008 the Zambian government and Ministry of Health (MOH) recruited a diverse multidisciplinary team of Zambian policymakers, Ipas and WHO representatives to participate in an assessment on unsafe abortion based on the WHO Strategic Assessment approach to strengthening reproductive health services [36]. Members of the strategic assessment team are responsible for maintaining continuity in the process that begins with the assessment and leads to testing interventions and scaling-up policies. Team members came from a range of organizations and reflected a diversity of perspectives on issues related to abortion and reproductive health, including, University of Zambia Senior Lecturers; programme managers; service-delivery providers; health and social science researchers; women’s health advocates; and external facilitators with experience using the WHO Strategic Assessment Approach.

Findings of the Strategic Assessment concluded that because gynecologists are only available at general and central hospitals, many women from rural areas must travel great distances to access reproductive health services [21]. Negative staff attitudes toward abortion further discouraged women from seeking safe services even in the limited places where these services were presumably available. These issues are exacerbated amongst adolescents, who face additional stigma accessing safe abortion, lack of areas in facilities specifically for adolescents, and lack of information on services [2, 24, 31, 37].

The recommendations stemming from the strategic assessment called for the scale-up of medical abortion and the training of midlevel providers for MVA provision to improve access and availability as well as the standard of care and called for better education and training programs on abortion for the health workforce. Additionally, participants saw the need for improved understanding of the public on their rights regarding abortion. Finally, they noted that the lack of national guidelines, which were finally drafted for the first time in 2009, on abortion hindered progress on scale-up of safe abortion across the country [21].

## Methods and scaling up of medical abortion in Zambia

In response to the findings of the Strategic Assessment, a collaborative intervention between the MOH, UTH and Ipas was established with funding from the Consortium for Research on Unsafe Abortion in Africa with the aim of introducing medical abortion in Zambia for the first time while addressing the multiple concerns of the Strategic Assessment team. An implementation science model was proposed to evaluate and determine effectiveness as well as gleaning lessons learned about bringing safe and legal abortion services to scale. The intervention involved the provision of CAC services in 28 public health facilities in Zambia for a 2-year period, from August 2009 to September 2011. The study focused on three main areas: building health worker capacity to provide PAC and induced abortion through the first 14 weeks of pregnancy in public sector facilities and introducing abortion with medication, working with pharmacists to provide improved information and access to MA, and community engagement and mobilization to increase knowledge of abortion service availability and rights among the general public through stronger health system and community partnerships. The research was reviewed and approved by the University of Zambia Biomedical Research Ethics Committee on 10 August, 2009.

**Fig. 1:**
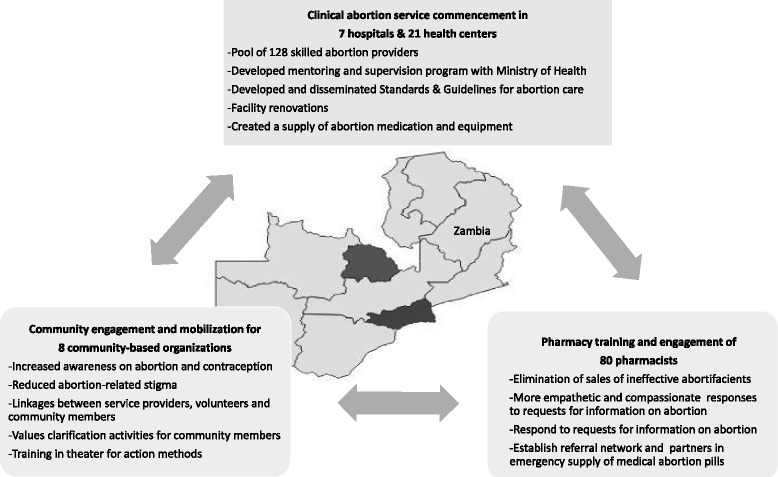
Integrated components of the Zambia comprehensive abortion care implementation science introduction and scale up

The health services package of activities included development and distribution of national standards and guidelines for safe abortion and the reduction of unsafe abortion, provider training in CAC, mentoring and supervision of providers, site renovations, registration of a medical abortion product, and the provision of MVA and medical abortion in 28 facilities of Lusaka and the Copperbelt. Health care workers were trained to provide induced abortion with either MVA or the combined product of 200 mg of mifepristone orally at the facility and 800 mcg of misoprostol 24–48 h later, either by returning to the facility or at home, depending on her preference and to treat incomplete abortion with MVA. Additionally, project staff worked with partners to build the capacity of the Planned Parenthood Association of Zambia (PPAZ), Youth Vision Zambia, Society for Women and Aids in Zambia, and Women in Law and Development in Africa and Women and Law in Southern Africa to create awareness about the law and services among their membership and reduce stigma in communities. The purpose was to create an enabling environment, provide more accurate information, appropriate referrals and greater community awareness to build support for access to sexual and reproductive health and rights, including safe abortion care. Figure 1 provides additional details on the three areas of intervention.

### Building health facility capacity for comprehensive abortion care

Health system capacity building began with improvement of abortion clinical services in 7 hospitals and 21 primary health care facilities in Lusaka and Copperbelt Provinces, the two most populous provinces in Zambia. Ipas and government partners trained 128 providers (including 4 mentors) at the 28 sites to provide treatment for unsafe abortions (PAC) and to provide safe and legal abortion services at low or no cost, similar to contraception and other maternal health services in the public sector. Implementers established a mentoring and supervision program for providers for the 1.5 years following their training. They also developed, published and distributed standards and guidelines on reducing maternal mortality and unsafe abortion. To improve access to supplies, the project assisted with the registration of mifepristone products in the country, provided MVA and MA supplies to each site and began to negotiate and advocate for the creation of a sustainable supply of abortion technologies in the private and public sectors by identifying and purchasing from a local private distributor. Additionally, during this time, another international NGO began quietly providing induced abortions to Zambian women who could afford them in a small number of urban areas in the country.

#### Working with pharmacists

The second phase of the implementation science project involved working with pharmacists who may encounter women seeking information on medical abortion as described in Fetters et al. [38] Although mifepristone and misoprostol are provided with prescriptions only, 80 pharmacists were trained and given updated information on medical abortion, technical updates and community referral systems were developed to increase linkages and combat medication stock-outs in their role as gatekeepers who frequently receive requests from both women and health care workers for abortifacients and information during an unplanned pregnancy. Pharmacy workers were trained to respond to clients seeking information on unplanned pregnancy in a more compassionate manner, to provide more accurate information on regimens and use of MA, and to provide referral information to women seeking safe abortion services in a participating health facility.

#### Community mobilization and engagement

Working with PPAZ, along with seven purposively selected civil society community-based organizations (CBOs), staff and volunteers conducted outreach activities to inform their membership and volunteers about contraception, the abortion law and services and to reduce related stigma by speaking more openly about the need for reproductive choices in Zambia. The CBOs used their own expertise to develop the new information into low literacy printed materials, flip charts for small group interactions and scripts for radio and community theater projects, amongst other activities. The project also created linkages between CBOs and service providers to increase their interaction, bringing community and health care workers together for values clarifying exercises to provide opportunities for them to reflect together on personal moral dilemmas about abortion.

#### Intervention evaluation

Program monitoring and evaluation was conducted for each intervention component – health services, pharmacy and community - using a range of process and outcome measures and methodologies listed in Table 1 and disseminated in an interactive workshop in Zambia in 2012 [39]. Findings were synthesized and discussed among more than 150 project stakeholders and policymakers to glean important lessons on the successes and challenges of MA/MVA provision and scale-up in Zambia and improve the sustainability of the service introduction for movement from the implementation science pilot to a full scale national program.

**Table 1:** Intervention components and evaluation strategies

Strategic component	Type and frequency of monitoring and evaluation
Health systems	Summary service statistic data from logbooks abstracted for each abortion and PAC procedure
	Facility assessments with key informants completed at baseline and endline at each facility
	Individual provider interviews (*N* = 104) at baseline and endline
	Individual client exit interviews at endline: 906 immediately following the MVA procedure or the initiation of MA and 356 at follow-up visit 7–10 days afterwards
Pharmacy orientation	Individual baseline and endline pharmacy interviews, 55 before training and 53 at 12–18 months post-training
	Baseline and endline mystery client visits to pharmacies at each participating pharmacy at baseline (*N* = 76) and at endline (*N* = 85)
Community engagement and mobilization	Quarterly activity reports from each of the eight CBOs reaching more than 36,000 community members
	Randomized household surveys with 568 respondents at baseline and 744 respondents at endline

## Discussion

### Scale up of services

**Fig. 2:**
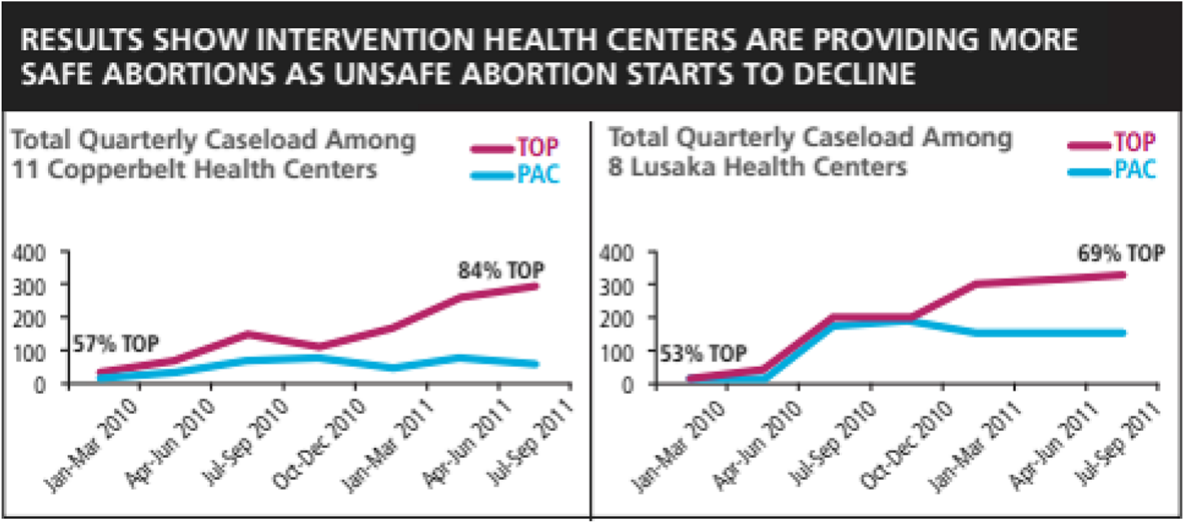
Increases in pregnancy terminations as treatment for unsafe abortion complications and miscarriages begin to decrease in intervention health centers

The first and most salient success of the scale-up effort was the reach of MA/MVA services to women in Zambia. After two years, 25 of 28 sites continued to provide CAC services, providing more than 13,000 women with abortion care during 21 months of data collection. Despite repeated attempts to engage managers and requests to train providers, three of the sites remained unsupportive of introducing the services or could not identify a provider willing to be trained to provide abortion care. Twenty percent of clients received induced abortions (2,766) while 80% were treated for complications of an unsafe abortion or miscarriage (10,881). More than half, 55% of all procedures (*N* = 7,470), were performed at the University Teaching Hospital, 26% (*N* = 3,574) in other hospitals and 19% (*N* = 2,616 cases) were performed in health centers, the first time abortion care has been decentralized outside of the nation’s hospitals. Prior to the intervention several hospitals provided some PAC services, most provided no induced abortions; none of the health centers provided any abortion care prior to this introduction, often forcing women to travel vast distances and delaying appropriate care. Most induced abortions (78%) were performed with mifepristone and misoprostol with the remaining using MVA (14%) or misoprostol alone (8%). The scale up in services resulted in a notable increase in safe abortions over time; particularly in health centers, which showed a levelling off or slight decline in PAC procedures (for complications of unsafe abortion and miscarriages) as safe abortions in these communities increased (Fig. 2).

#### Improved provider attitudes and capacity

At the end of the intervention, all of the providing facilities (*N* = 25) had management that was supportive of continuing legal abortion services as reported by the providers themselves. The providers trained as part of the intervention who were interviewed at endline (*N* = 104) were midwives (*N* = 43), clinical officers (*N* = 22), nurses (*N* = 21), physicians (*N* = 16) or other types of providers (*N* = 2). During short in-person interviews, providers were asked a series of multiple choice and short answer questions; written notes were taken verbatim on the open-ended questions. Text from open-ended questions were transcribed and manually organized thematically for analysis by the study personnel. Almost two-thirds reported being “extremely confident” using MVA. Providers reported themselves being “very comfortable” in counseling on abortion (87%) and providing abortion (67%). Most providers (61%) perceived the abortion law in Zambia as appropriate while 21% described it as too restrictive – only 14% described it as too liberal. All providers agreed that women should have access to PAC and almost all (99%) agreed that women should have access to induced abortion. A majority of providers had spoken to someone outside of the clinic about abortion decisions (77%); had talked with a colleague in the facility about their provision of abortion care (97%); had worked in their communities to inform women about safe abortion services (51%); and had told a family member (80%) or friend (92%) that they provide induced abortion services, thus leaving a newly experienced and more supportive foundation for expanded abortion care in the country where women often seek the informal advice of health workers before seeking care [7].

When asked specifically about the impact of MA provision on their services, a number of providers reported that MA improved their ability to provide affordable, safe abortion care for their clients.

*[MA] has improved the TOP services because clients are always assured of finding the drugs here at the facility and are not forced to buy at expensive prices.* – male, clinical officer, 2009

MA provision has also increased providers’ confidence and their ability to maintain client safety and confidentiality.

*[MA] has made providers more confident. MVA started earlier than MA at this facility, but since we started [MA], we have been having less complicated cases. Confidentiality is maintained because clients do not need to go from place to place in the facility.* – male, clinical officer, 2009

#### Pharmacists as partners in MA provision

Regarding their attitudes toward abortion, at the end of the study, all pharmacists and pharmacy workers who participated in the intervention and were interviewed agreed that they should provide information on safe abortion to women who request it. Nearly all agreed that healthcare workers deserve respect for providing abortion care (98%) and that abortion should be legally available in Zambia for any woman who needs it (83%). Among those interviewed at endline, 96% reported discussing MA with other staff at the pharmacy since their training and 84% had spoken to a friend or relative about the risks and benefits of having an abortion. More than two-thirds (68%) reported recommending that a patient seeking an abortion visit a facility with safe services [38, 40].

Overall, more pharmacists and pharmacy workers offered to sell MA or gave information about MA after the intervention, increasing from 46% in 2009 to 66% of mystery client interactions in 2011. Mystery clients also reported that 41% of pharmacy workers who did not offer to help at endline visits were at least sympathetic to their problem, compared with only 15% at the baseline visits. Only 19% of mystery clients were offered misoprostol for purchase at baseline, but this increased to 47% after the intervention. The full results of the pharmacy evaluation are reported elsewhere [38, 40].

#### Increased support for safe and legal abortion at the policy level

Since the conclusion and dissemination of the evaluation results, bilateral funding for Zambia from two anonymous donors has been secured for 6 years, up to 2017, and CAC services have been introduced in 84 additional facilities, creating more decentralized services and improving access to CAC across the country that now provides more than 10,000 safe and legal abortion procedures each year [41]. Four tertiary hospitals now provide CAC services and use updated clinical protocols for training physicians. There is more research, including over six peer-reviewed articles on abortion in Zambia, and more attention and watchful examination of safe abortion issues on a national level, with the subsequent media training in abortion and SRH issues of over 50 local journalists [42–45]. Twenty-five of the original 28 original intervention sites are still providing care where services are technically provided free or at low cost.

### Ongoing challenges

#### Provider and community stigma

Despite progress in improved attitudes, such as empathy and concern, towards abortion clients and improved community engagement, the evaluation revealed continuing stigma on both the provider and client side. The majority of trained providers expressed personal support for induced abortion; however, 80% of the facilities where they worked still had personnel who they felt would oppose an expansion of abortion services or training. Only 12 (48%) of the facilities had a sign posted to declare that abortion care was available and just 11 (44%) of the facilities had the same intake procedures for abortion as for other maternal and child health services, suggesting that after the intervention abortion was still being treated as a different and more shameful kind of service than other medical care.

Yet there have been legal barriers, including arrests of abortion providers, clients and a lengthy ban on the provision of abortion care by an international nongovernmental organization reported to have bypassed the requisite authorizations to perform abortion services since the time of the study [46]. The overall fragile and political nature of this work was highlighted by the shut-down of activities of an international NGO in 2012 due to the initiative of a single member of parliament [46]. Currently, professional associations are rallying against strong opposition as Parliament reviews the Constitution and considers a referendum stating that “life begins at conception”. These tactics further highlight the restrictive nature of abortion consent laws, which do not serve to improve women’s access to comprehensive reproductive care.

Before the intervention, individuals surveyed in community households demonstrated a lack of knowledge about and support for legal abortion [47]. Respondents often expressed strong beliefs about the immorality of abortion while concomitantly professing a belief in a woman’s right to choose an abortion, eliciting the complex nature of this issue for many people. After the intervention, more respondents were aware of the legality of abortion but fewer believed that women should have access to safe abortion services. That said, the proportion of community members who understood the complicated legal indications for pregnancy termination remained low.

#### Erratic drug supply

Early in the study, the supply of mifepristone to the country was erratic and stock outs were common, as questions about the new drug arose from procurement, MOH, and hospital administrators. Currently, as many as five mifepristone products are registered for use in Zambia but price reductions and cost-sharing contributions from the Government and bilateral donors have floundered, even as the need for these drugs continues to increase. Maintaining a consistent supply chain for mifepristone and establishing national price agreements is essential to ensuring consistent service availability as more women learn about and seek out MA services. However, MA distribution during the intervention improved networking and collaboration among facilities and pharmacies; to avoid stock-outs hospitals became drop-off and distribution points for drugs and took on a mentoring and on-site training roles to nearby health centers.

#### Human resources and health system constraints

While substantial progress was made during the implementation of scale-up, systemic resource constraints in the health system hampered efficient and effective delivery of CAC and PAC services to women. Substantial proportions of the facilities reported sometimes having to turn patients seeking PAC (20%) or abortion services (60%) away because of lack of a trained provider on duty. As noted above, chronic shortages in basic and specific medical supplies also hampered timely delivery of quality care.

## Conclusions and lessons learned

Perhaps the first and most critical lesson learned from this case study is the importance of engagement and support from local policy and implementation stakeholders. The impetus for the current efforts on CAC stemmed from national-level awareness of the impact of unsafe abortion on the maternal mortality rate and the increased global attention brought to this issue by the MDGs. This awareness presented an opportunity for partnerships, which resulted in both the strategic assessment on unsafe abortion and the subsequent implementation science study on the scale-up of CAC services. Global efforts, such as the MDGs, WHO global policy and technical updates, and the Sustainable Development Goals (SDGs) should be utilized to catalyze national efforts and interest in advancing abortion care and eliminating unsafe abortion. The strategic assessment itself united multiple local stakeholders and provided critical evidence in support of MA scale-up, which further spurred stakeholders to action. Without support from the MOH and local stakeholders, provision and scale-up of induced abortion services would have been impossible even after the evaluation findings.

Although the intervention was focused on induced abortion, the introduction and improvement of PAC services was an important extra benefit. Before the intervention, only four of the hospitals and two of the health centers provided PAC, and only the UTH provided induced abortion in any systematic way. While most PAC cases were treated in hospitals throughout the study, the intervention succeeded in expanding PAC to health centers – closer to where women live – in an effort to decentralize this important service and allow over-crowded hospital rooms to treat serious cases more efficiently. Introducing misoprostol for PAC in health centers could help in further decentralizing PAC to the health center level but by far the most important factor would be a reconsideration of guidelines to allow midlevel providers to train and provide CAC services without physician signatures. An expansion of the scope of MLPS in the provision of CAC would allow for the expansion of services to the most remote areas of the country and potentially improve contraceptive acceptance while decreasing unintended pregnancies and repeat abortions.

The study demonstrated that trained mid-level providers can deliver safe abortion services. However, values clarification appeared necessary to change attitudes among many groups (e.g., providers, facility managers, the general public, pharmacists and pharmacy workers) to improve abortion care and increase awareness of these services. Health workers still face opposition in their facilities and the need remains for management to create an environment more supportive about abortion care. Additionally, more open discussion about both safe and unsafe abortion at the community level, through volunteers and mass media, will continue to normalize abortion care and place it in the context of the package of essential maternal health care. Additional efforts are needed to support and normalize abortion care services and integrate them into the rest of the public health system.

The study results demonstrated that some pharmacists and pharmacy workers have a role to play in safe abortion services and are willing to play it. The mystery client interactions show an increase in pharmacists and pharmacy workers’ willingness to provide information, drugs for MA, and health facility referrals over the 2 years of the study. Mystery clients also reported fewer hostile interactions and increased sympathy from pharmacists and pharmacy workers.

Contraception plays a crucial role in reducing the need for abortion services, and almost half of women who received abortion care left with a contraceptive method. Postabortion contraceptive acceptance was better at health centers than at hospitals; however, contraceptive counseling and method provision should be better integrated into all abortion services. If these women had tried to induce their abortions on their own or had gone instead to unsafe providers, it is unlikely that they would have been counseled for contraception or have received a method. However, when asked directly after their procedures, there were still women who wanted a method who left the facility without one and many who received a method reported “wanting to learn more” about family planning.

At endline, many respondents did not know that abortion was legal and responded negatively to questions about their attitudes toward abortion. The lack of clarity and communication about the national abortion law and policies serves only to confuse the populace and prevent or delay women from seeking services to which they are legally entitled. The community engagement and mobilization component of the intervention improved community members’ knowledge about the legal abortion services available but had less impact in changing feelings and deep-rooted stigma about abortion services. Further efforts must be made to inform women of their right to and availability of safe abortion and to change community perceptions about this highly stigmatized topic.

Finally, to maintain and continue to make progress on access to safe abortion, there is a need for a broader coalition of partners to build support. This should include the MOH but not be limited to them. Champions for this project in the MOH have been highly mobile and have often moved to positions where they lose their ability to speak freely on sensitive topics. By creating a more robust base of support among policymakers, the safe abortion movement will not be as vulnerable to frequent political reappointments. Furthermore, there is a continued need to build a vertical but integrated CAC service within other maternal health or gynecological services. Placing abortion firmly within the constellation of safe and legal reproductive health services is the only means by which to ensure improved access for all women.

The experience of introducing MA and scaling up safe abortion in Zambia offers important lessons for other countries seeking to improve maternal outcomes by improving access to comprehensive abortion care. The Zambian experience can provide an avenue for safe abortion services in other contexts where abortion rights are provisionally legal but practically restrictive. By building local support among stakeholders, coordinating efforts of implementers and using evidence to guide and inform decision-making, study partners were able to scale-up access to abortion services in an otherwise barren landscape of care. However, as the results of the evaluation show, there is still much work to be done to ensure that the level of care remains high, that access across the country continues to grow, and that stigma for abortion services, be jettisoned in favor of putting women’s lives first.

### Data sharing

The original data in the form [45] of anonymized survey results and anonymized and aggregate service statistics are available from TF in an excel spreadsheet and Stata 11 data sets.

## Abbreviations

CAC: Comprehensive abortion care
CBO: Community-based organization
MA: Medical abortion
MDGs: Millennium development goals
MMR: Maternal mortality ratio
MOH: Ministry of health
MVA: Manual vacuum aspiration
NGO: Non-governmental organization
PAC: Postabortion care
PPAZ: Planned parenthood of Zambia
TOP: Termination of pregnancy
UTH: University teaching hospital
WHO: World Health Organization
